# Novel strong promoter of antimicrobial peptides gene *pro-SmAMP2* from chickweed (*Stellaria media*)

**DOI:** 10.1186/s12896-016-0273-x

**Published:** 2016-05-18

**Authors:** Roman A. Komakhin, Denis A. Vysotskii, Rahim R. Shukurov, Vera D. Voblikova, Vera V. Komakhina, Svetlana R. Strelnikova, Ekaterina M. Vetchinkina, Alexey V. Babakov

**Affiliations:** All-Russia Research Institute of Agricultural Biotechnology, Timiriazevskaya 42, 127550 Moscow, Russia; IBC Generium LLC, Vladimirskaya 14, 601125 Volginsky, Russia

**Keywords:** *Stellaria media*, *Nicotiana tabacum*, *pro-SmAMP2*, Promoter, Expression control, Transgenic plants

## Abstract

**Background:**

In a previous study we found that in chickweed the expression level of the *pro-SmAMP2* gene was comparable or even higher to that of the β-actin gene. This high level of the gene expression has attracted our attention as an opportunity for the identification of novel strong promoters of plant origin, which could find its application in plant biotechnology. Therefore, in the present study we focused on the nucleotide sequence identification and the functional characteristics of the *pro-SmAMP2* promoter in transgenic plants.

**Results:**

In chickweed (*Stellaria media*), a 2120 bp promoter region of the *pro-SmAMP2* gene encoding antifungal peptides was sequenced. Six 5′-deletion variants −2120, −1504, −1149, −822, −455, and −290 bp of *pro-SmAMP2* gene promoter were fused with the coding region of the reporter gene *gusA* in the plant expression vector pCambia1381Z. Independent transgenic plants of tobacco *Nicotiana tabacum* were obtained with each genetic structure. GUS protein activity assay in extracts from transgenic plants showed that all deletion variants of the promoter, except −290 bp, expressed the *gusA* gene. In most transgenic plants, the GUS activity level was comparable or higher than in plants with the viral promoter *CaMV 35S*. GUS activity remains high in progenies and its level correlates positively with the amount of *gusA* gene mRNA in T_3_ homozygous plants. The activity of the *рro-SmAMP2* promoter was detected in all organs of the transgenic plants studied, during meiosis and in pollen as well.

**Conclusion:**

Our results show that the *рro-SmAMP2* promoter can be used for target genes expression control in transgenic plants.

**Electronic supplementary material:**

The online version of this article (doi:10.1186/s12896-016-0273-x) contains supplementary material, which is available to authorized users.

## Background

Currently, promoters of many genes from a wide range of organisms are used in genetic engineering of plants. Promoters are traditionally divided into three types: constitutive, tissue-specific, and inducible [1]. In former times, virtually all transgenic plants contained two heterologous genes: one, a selection marker gene controlled by a constitutive promoter and the other a target gene controlled by a promoter of any type to change the plant phenotype. At present, multigene transformation allows the import of entire metabolic pathways into plants, including expression of protein complexes consisting of several target genes for the generation of transgenic plants producing multiple compounds simultaneously [2, 3]. Either a number of different promoters with similar level and profile of expression, or several copies of the same promoter must be used in one genetic construct for multigene transformation. Both approaches are complicated for a number of reasons. Firstly, there is a lack of available promoters with the necessary parameters. Secondly, the introduction of repetitive sequences in the same or different loci of a transgenic plant genome can have a negative effect on the expression and inheritance of heterologous genes due to the effect of homology-dependent gene silencing [4–6].

Up to now, the constitutive promoter *CaMV 35S*, created on the basis of shell protein 35S gene promoter region from cauliflower mosaic virus CaMV, is most widely used in vectors for genetic transformation of plants [1, 7, 8]. The *CaMV 35S* promoter usually provides a high expression level of heterologous genes in plants, but it has some significant drawbacks. The promoter originates from a virus and infection of transgenic plants with CaMV virus may lead to the silencing of a heterologous gene controlled by *CaMV 35S* [9]. In addition, the widespread use of the *CaMV 35S* promoter increases the probability to inactivate expression of heterologous genes under its control as a result of the homologous recombination [10, 11].

In addition to *CaMV 35S*, a number of other viral promoters are used for plant biotechnology (see the review by Porto et al. [8]). Recently, a highly efficient promoter was created from individual parts of different viral promoters [12, 13]. Such a widespread use of viral promoters is in part due to the lack of well-characterized strong plant gene promoters, particularly from dicotyledons.

For monocotyledonous plants, strong promoters are known, such as *Act1* and *ZmUbi*, which are already used in crop plant genetic engineering for quite a long time [14–16]. Recently, several strong and constitutive promoters *APX*, *PGD1* and *R1G1B* from rice (*Oryza sativa*) were proposed for biotechnology of the same plant, which performed significantly better than the previously studied promoters *Act1* and *ZmUbi* [17]. Strong and constitutive promoters of ubiquitin genes *RUB2* from *O. sativa* and *UBI10* from *Brachypodium distachyon* were characterized and demonstrated to exceed *CaMV 35S* in activity up to 35-fold in transgenic monocotyledonous plants [18, 19].

At the same time, a number of strong and constitutive promoters were cloned for genetic engineering of dicotyledonous plants, but they not perform as well as those from the monocots described above. The promoters of *UBQ1* and *UBQ6* genes from *Arabidopsis thaliana* are active in all tissues of tobacco (*Nicotiana tabacum*) at the level of viral *CaMV 35S* promoter [20]. Quantification of promoter strength using transient expression in lima bean (*Phaseolus lunatus*) cotyledonary tissue showed that *Gmubi* and *GmScream* promoters from soybean (*Glycine max* Merr.) yielded from 2 to 7-fold higher expression than a standard *CaMV 35S* promoter [21, 22]. The promoter of the acetolactate synthase *ALS3* gene from cabbage (*Brassica oleracea*) is constitutive and comparable in efficiency to *CaMV 35S* [23]. Use of the strong constitutive promoter *MtHP* from *Medicago truncatula* to drive *gus* expression in *Arabidopsis*, showed around 50 % higher GUS activity as compared to the *CaMV 35S* promoter. The activity of this promoter in transgenic plants of clover (*Trifolium repens*) and alfalfa (*Medicago sativa*) was 1.75 and 1.4 times higher than that of *CaMV 35S*, respectively [24]. The promoter of ACC-synthase *VR-ACS1* gene from mung bean (*Vigna radiata* L.) demonstrated greater efficiency producing up to 6 times higher activity of the reporter proteins in transgenic tobacco and *Arabidopsis*, compared to the *CaMV 35S* [25]. The apparent superiority of the *VR-ACS1* promoter over the viral one was not only the result of transcriptional activation but also of more efficient translation.

Despite the abundance of literature on the use of plant promoters in plant biotechnology, the lack of effective promoters for high expression of target genes still exists.

In this study we focused on another group of plant genes that can be a source of effective promoters. When studying antifungal peptides of chickweed (*Stellaria media*), we found that the expression of their genes *pro-SmAMP1* and *pro-SmAMP2* is high and that it increases when *S. media* is treated by the elicitor methyl jasmonate or infected by pathogenic fungi [26]. The *pro-SmAMP1* gene expression increased from 10 to 70 times reaching the level of the chickweed house-keeping gene β-actin. The expression of the other gene, *pro-SmAMP2*, is rather constitutive because its activity does not change so much under the influence of the above factors (2–5 times). At the same time its normal level of activity was unusually high in different organs (stem, leaves, roots, flowers), comparable or superior to the level of β-actin expression.

The high expression level of *pro-SmAMP2* gene indicates that this gene has a strong promoter, which may be of interest for application in plant biotechnology. For that reason, we identified the promoter sequence and studied the properties of the *pro-SmAMP2* gene promoter.

## Results

### Cloning the nucleotide sequence of *pro-SmAMP2* gene promoter region and production of genetic constructs for plant transformation

**Fig. 1:**
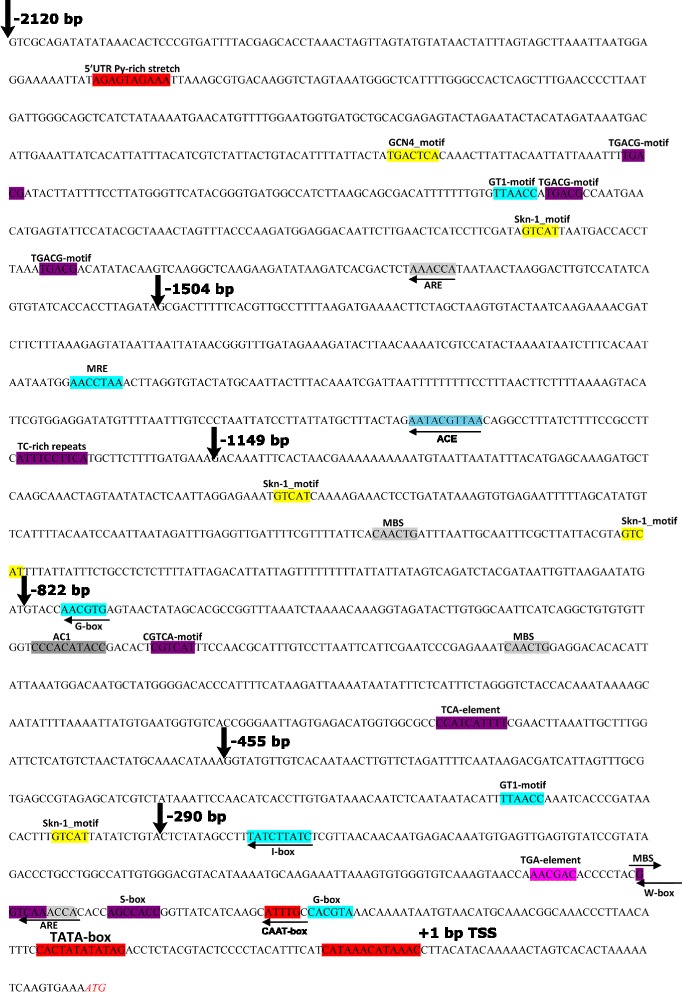
The nucleotide sequence of 5′-flanking promoter region of *pro-SmAMP2* gene and location of *cis*-acting elements (color-coded and labeled). Vertical arrows mark the starting points of the 5′-deletion variant nucleotide sequences. TSS is the transcription start site at −40 bp from ATG codon behind the region highlighted in red. Translation initiation site ATG +41 is labeled in italics

As a result of “genome walking”, a 2400 bp target fragment of *S. media* genome was amplified. Its sequencing showed that the fragment includes part of the coding sequence of the *pro-SmAMP2* gene and 2160 bp 5′-flanking region from the translation start site (ATG codon) including 40 bp of *pro-SmAMP2* gene 5′-UTR. The nucleotide sequence including the expected promoter region was analyzed by programs PLACE [27, 28] and PlantCARE [29], which revealed some regulatory motifs previously found in most eukaryotic promoters (Fig. 1 and Table 1). For instance, the following sequence features were found: one W box, four TGACG motifs, one S box, four binding sites of MYB transcription factor, TC-rich repeat, one TGA-element involved in response to auxin and one ARE element involved in response to anaerobic conditions. In addition, motifs responsible for tissue-specific gene expression or expression that is invoked in response to the light conditions are present in the nucleotide sequence of the promoter region. Amongst these, were one GCN4 motif and four Skn-1 motifs, both of which are associated with increased expression in the endosperm of seeds, one AC-I motif associated with increased expression in the xylem, one I box, two GT1 motifs and two G box elements, all related to LRE elements responsible for expression in above-ground parts and regulated by light exposure. The presence of such motifs suggests that the activity of the *pro-SmAMP2* gene in chickweed plant can be regulated by the interaction of different transcription factors with the corresponding *cis*-elements within the promoter. Moreover, the *pro-SmAMP2* gene promoter contains the conservative sequence of TATA-box CATTTCCACTATATATAG, CAAT-box motif and the transcription initiation site CAN(A/C)(A/C)(C/A)C(C/A)N_2_A(C/A). Altogether, this analysis suggests that *pro-SmAMP2* promoter is strong and potentially regulated in response to stress, as for example, pathogen attack and/or light exposure.

**Table 1:** Motifs detected in *pro-SmAMP2* gene promoter sequence (based on the results of analysis by PLACE and PlantCARE programs)

Motif	Description
5UTR Py-rich stretch	region in the 5primeUTR conferring high transcription levels without the need for other upstream *cis*-elements except for a TATA-box
GCN4_motif	*cis*-regulatory element involved in endosperm expression
TGACG-motif, CGTCA-motif	*cis*-acting regulatory element involved in the MeJA-responsiveness
GT1-motif	light responsive element
ARE (anaerobic responsive element)	*cis*-acting regulatory element essential for the anaerobic induction
MRE	MYB binding site involved in light responsiveness
ACE	*cis*-acting element involved in light responsiveness
TC-rich repeats	*cis*-acting element involved in defense and stress responsiveness
Skn-1_motif	*cis*-acting regulatory element required for endosperm expression
MBS	MYB binding site involved in drought-inducibility
G-box	*cis*-acting regulatory element involved in light responsiveness
AC-I	element involved in negative regulation on phloem expression; and responsible for restricting the vascular expression to the xylem
TCA-element	*cis*-acting element involved in salicylic acid responsiveness
I box	part of a light responsive element
TGA-element	auxin-responsive element
W-box	wounding and pathogen elicitor response
S box	wounding and pathogen elicitor response
CAAT-box	common *cis*-acting element in promoter and enhancer regions
TATA-box	core promoter element around −30 of transcription start

The sequence of the *pro-SmAMP2* promoter region shown in Fig. 1 also includes the 5′-UTR of *pro-SmAMP2* gene, which was only 40 bp in the 5′ direction from the translation initiation site ATG.

**Fig. 2:**
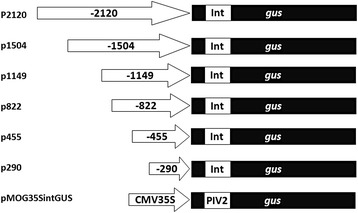
The scheme of constructs with deletion variants of *pro-SmAMP2* gene promoter region designed on basis of plant expression vector pCambia 1381Z for analysis of *gus* reporter gene expression. Plant expression vector pMOG-35SintGUS containing *CaMV 35S* viral promoter was used as a control. The translated region of *gus* gene is colored black; Int - modified castor bean catalase intron within the translated region of *gus* gene; PIV2 - modified potato *ST-LS1* gene intron within the translated region of *gus* gene. Promoters are depicted as arrows with the appropriate signatures

In order to study the properties of the *pro-SmAMP2* gene promoter related to the expression of genes in heterologous systems, six genetic constructs, containing various 5′-deletion variants of the promoter region with 5′UTR, were obtained (see Methods and Fig. 2).

Deletion variants of the *pro-SmAMP2* promoter region were chosen in such a way that the location of *cis*-regulatory elements in the nucleotide sequence was taken into account. The plasmid pMOG-35SintGus, in which the *gus* gene is controlled by the *CaMV 35S* viral promoter, was used as a positive control. In the vectors, modified castor bean catalase intron or *ST-LS1* gene intron was placed within the sequence of the *gus* gene (Fig. 2) to prevent the influence of *Agrobacterium* contamination on the results of GUS activity measurements in transformed plants.

### *Pro-smAMP2* promoter yields higher GUS activity in T_0_ transgenic tobacco plants than the *CaMV 35S* promoter

After *Agrobacterium*-mediated transformation, independent T_0_ tobacco plants, each taken from individual calli, were selected in the following number: T_0_p2120 - 10 plants, T_0_p1504 - 9 plants, T_0_p1149 - 12 plants, T_0_p822 - 13 plants, T_0_p455 - 8 plants, T_0_p290 - 22 plants, and control Т_0_pMOG-35SintGus - 15 plants. Estimation of the GUS activity in protein extracts from leaves of aseptic transgenic plants showed that all deletion variants of the *pro-SmAMP2* promoter except −290 bp (Additional file 1: Table S1) expressed the *gus* gene.

**Fig. 3:**
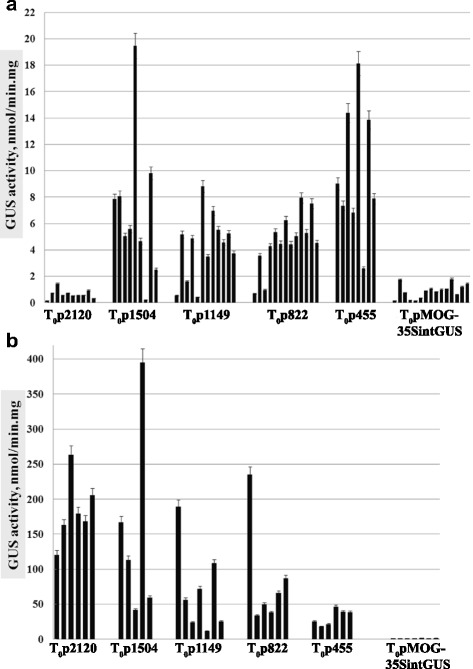
The GUS activity in tobacco transformants T_0_. **a** Plants were growing under aseptic conditions 1.5 months after rooting on the medium with a selective agent. The average values of activity from three mature leaves of each transformant ± SE (the standard error) are presented. **b** Plants were grown for six weeks in soil after being transplanted from aseptic conditions. The average activity values of three samples from one leaf of each transformant ± SE are presented

In most transgenic plants from groups T_0_p1504, T_0_p1149, T_0_p822 and T_0_p455, the GUS activity ranged from 2 to 20 nmol/mg · min. This was several times higher than the GUS activity measured in the control plants Т_0_pMOG-35SintGus, ranging from 0.2 to 1.8 nmol/mg · min (Fig. 3a). The level of GUS activity in T_0_p2120 plants was comparable to plants expressing *gus* under the control of *CaMV 35S* promoter, and did not exceed 1.8 nmol/mg · min (Fig. 3a). Differences in GUS activity levels amongst independent primary transformants of different constructs is most probably due to the integration of multiple T-DNA copies into their genome or chimerism.

Similar to the measurements in aseptic plants, GUS enzymatic activity was analyzed in the tobacco leaves of T_0_ plants, grown in soil. First, Fig. 3b shows that the maximum levels of GUS activity in leaves of T_0_ transgenic tobacco plants grown in the soil were notably higher than the values obtained for the same plants under aseptic conditions. On average, GUS activity values of 180 nmol/mg · min in T_0_p2120, 150 nmol/mg · min in T_0_p1504, 70 nmol/mg · min in T_0_p1149, 84 nmol/mg · min in T_0_p822, and 31 nmol/mg · min in T_0_p455 were observed. However, a 5 to 10-fold difference was detected between some of the individual Т_0_p1504, Т_0_p1149 and Т_0_p822 plants. Secondly, unlike under aseptic conditions, in average, the highest GUS activity of plants grown in soil was observed in T_0_p2120 plants. Thirdly, decreasing the length of the *pro-SmAMP2* promoter resulted in a decrease in GUS activity. In the *CaMV 35S* plants the average GUS activity was not higher than 1.2 nmol/mg^**.**^min. It should be noted that the GUS activity of the Т_0_pMOG-35SintGus plants grown in soil was also slightly (1.5 to 2-fold) higher than in the aseptically grown plants.

The highest levels of GUS activity was measured in leaves of tobacco plants Т_0_p2120 (5 transformants), Т_0_p1504 (2 transformants), Т_0_p1149 (2 transformants), Т_0_p822 (2 transformants) and Т_0_p455 (5 transformants) growing in soil (Fig. 3b). GUS activity in the adventitious roots and flowers of the same plants was less than 20 % of the activity in leaves and ranged 2 to 5-fold between samples of one transgene version. GUS activity in stems of individual primary transformants Т_0_p455 (young sprouts 8–10 cm long) could be at the level of activity in leaves but it varied 2 to 4-fold from plant to plant. GUS activity in stems of other transgenic lines Т_0_p2120, Т_0_p1504, Т_0_p1149 and Т_0_p822 was not higher than 25 % of activity in leaves and it also varied greatly from plant to plant (Additional file 2: Fig. S1).

### *Pro-SmAMP2* promoter retains its activity in the progeny of T_1_-T_3_ transgenic plants

The level of GUS activity in the progeny of T_0_ transgenic plants was studied to characterize the properties of *pro-SmAMP2* promoter deletion variants across the generations. For that, seeds of T_1_ from five transgenic tobacco plants of each group Т_0_p2120*,* Т_0_p1504, Т_0_p1149, Т_0_p822 and Т_0_p455 expressing *gus* gene were produced by self-pollination. At least 200 seeds from each T_0_ plant representing one population were aseptically planted on selective medium containing the antibioticum hygromycin. After 4 weeks of selection, segregation of plants into the subpopulations of transgenic T_1_ (green) and non-transgenic (whitened) was analyzed. In some populations from groups T_1_p2120*,* T_1_p1504, T_1_p1149, T_1_p822 and T_1_p455, a segregation ratio of 3:1 between green and white plants was confirmed using the Chi-square criterion which is consistent with the presence of T-DNA at a single locus of the genome. These results, however, do not allow an estimate of the number of T-DNA repeats at such insertion locus.

In some T_1_ populations the segregation ratio was different from monogenic being equal to 2:1, probably as a result of elimination of one transgenic class. There were also T_1_ populations with a segregation ratio of 15:1, which suggests the presence of T-DNA in two unlinked loci of the ancestral T_0_ plant genome.

**Fig. 4:**
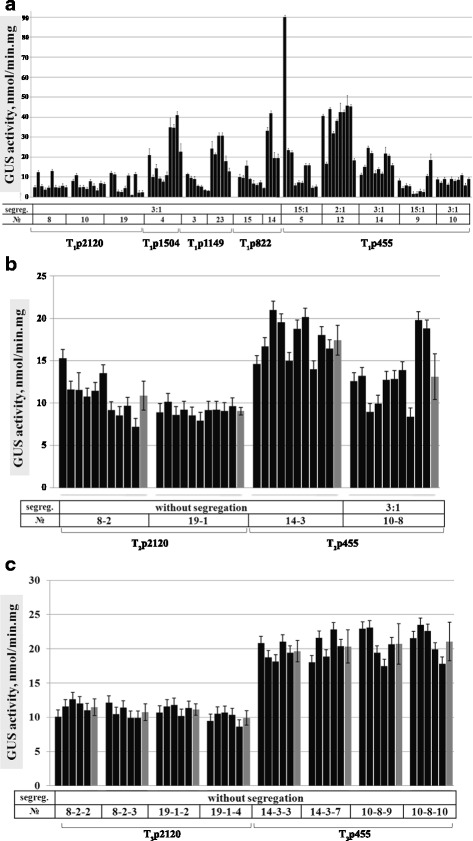
The GUS activity in leaves of transgenic tobacco plants from three consecutive generations (T1, T2, T3) measured in six weeks of growth in soil after transplanting from aseptic conditions. The average activity values of three samples from one leaf of each transformant ± SE are presented. The average activity among the plants of one population ± confidence interval (*p* = 5 %) are shown as gray bars. **a** T_1_. **b** T_2_. **c** T_3_

Enzymatic activity of GUS protein was studied in T_1_ plants with all deletion variants T_1_p2120, T_1_p1504, T_1_p1149, T_1_p822 and T_1_p455 to determine the level of the reporter gene *gus* expression. As shown in Fig. 4a, high GUS activity was still present in T_1_ transgenic tobacco plants carrying all deletion variants of the *pro-SmAMP2* promoter. Two-fold difference in levels of GUS activity between individual transgenic plants from the same T_1_ population with monogenic segregation observed in all variants most probably resulted from their T-DNA locus being homo- or heterozygotic (Fig. 4a). The difference in GUS activity between different populations with monogenic inheritance of T_1_p1149, T_1_p822 and T_1_p455 T-DNA could result from the integration of two T-DNA copies into one chromosome locus or from the positional effect of T-DNA insertion (Fig. 4a).

The GUS activity level was significantly lower in all T_1_ transgenic plants compared to the progenitor T_0_ plants. The highest reduction (more than 25-fold) was observed in the Т_1_р2120 plants. The activity decreased 8- to 11-fold in T_1_p1504, T_1_p1149, T_1_p822, and 3 to 5-fold in Т_1_p455 plants. Thus, the longer the nucleotide sequence of the promoter deletion variant, the greater the reduction in GUS activity in T_1_ plants compared to the progenitor T_0_ plants.

Nevertheless, even the level of GUS activity in transgenic T_1_ plants of the efficient deletion variants of the *pro-SmAMP2* promoter region was equal to or several times higher than the activity achieved with the *CaMV 35S* promoter (0.9-3.3 nmol/mg^**.**^min in [12, 30] and 10 nmol/mg^**.**^min in [25]).

For T_2_ generation studies, only transgenic plants with the longest −2120 bp and the shortest −455 bp deletion variants of *pro-SmAMP2* promoter were used. High GUS activity was preserved in transgenic tobacco plants of T_2_ generation with deletion variants −2120 and −455 bp of *pro-SmAMP2* promoter (Fig. 4b). In two independent Т_2_p2120 populations, No. 8–2 and No. 19–1, all transgenic plants were resistant to the selective agent hygromycin and did not differ significantly in the level of GUS activity from each other, what indicates that they are homozygous for the T-DNA locus. The average GUS activity level in the population No. 19–1 and No. 8–2 was 9.0 ± 0.4 nmol/mg^**.**^min and 10.9 ± 1.7 nmol/mg^**.**^min respectively, which corresponds to the activity in their parent T_1_ plants (Fig. 4a).

Unlike transgenic plants Т_2_p455 No. 14–3, all of which were resistant to hygromycin, the plant population No. 10–8 segregated in the ratio of 3:1 for resistance to hygromycin (Fig. 4b). These results show that plants of Т_2_p455 population No. 10–8 were obtained from T_1_ generation plants hemizygous for T-DNA locus. The average level of activity in plants of the population No. 14–3 was about 17.4 ± 1.8 nmol/mg · min, that is about 1.3 times higher than the average level in the population Т_2_p455 No. 10–8, being 13.1 ± 2.7 nmol/mg · min (Fig. 4b). At the same time, activity levels were close in plants Т_2_p455 No. 14–3 and in two plants from populations No. 10–8 with the highest activity, which, as we assume, were homozygous for T-DNA loci. In order to confirm this we produced and analyzed T_3_ generation. Two plants with GUS activity 2-fold higher than the other plants from the population Т_2_p455 No. 10–8 were used to generate T_3_ (Fig. 4b).

High GUS activity was preserved in T_3_ generation transgenic tobacco plants on the level of their parent plants T_2_ with *pro-SmAMP2* promoter deletion variants −2120 and −455 bp (Fig. 4b and с). All transgenic plants were resistant to hygromycin and had comparable level of GUS activity in individual plants, which suggested their homozygous state for T-DNA loci. The average level of GUS activity in T_3_p455 populations was as high as 19.6 ± 1.6 nmol/mg · min in No. 14-3-3, 20.3 ± 2.4 in No. 14-3-7, 20.7 ± 3.0 in No. 10-8-9 and 21.1 ± 2.8 in No. 10-8-10. The average level of GUS activity in T_3_p2120 populations was 11.5 ± 1.2 nmol/mg · min in No. 8-2-2, 10.8 ± 1.2 in No. 8-2-3, 11.1 ± 0.8 in No. 19-1-2 and 9.9 ± 1.1 in No. 19-1-10.

**Fig. 5:**
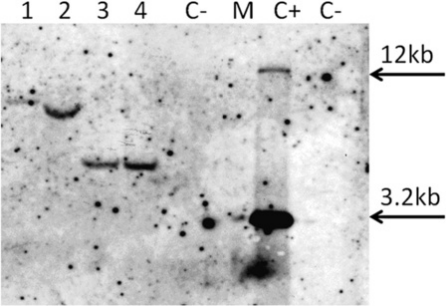
Southern blotting analysis of total DNA from leaves of two plants Т_3_p2120 No. 8-2-2 (lanes 1 and 2) and Т_3_p455 No. 14-3-3 (lanes 3 and 4) restricted with *EcoR*I. “C-“- total DNA from leaves of non-transgenic tobacco plants restricted with *EcoR*I. M - marker (Fermentas, #Sm333). “C+” - plasmid pMOG-35SintGus restricted with *Hind*III. The 740 bp fragment of *gus* gene was used as probe

Southern blotting analysis showed that Т_3_p2120 plants from population No. 8-2-2 and Т_3_p455 plants from population No. 14-3-3 contain T-DNA insertion at one genomic locus (Fig. 5). Consequently, GUS activity levels achieved in these plants was provided by a single locus T-DNA insertion.

### GUS activity changes during vegetative period in homozygous T_3_ lines with *pro-SmAMP2* promoter deletion variants −2120 and −455 bp under different light conditions

In previous experiments, the level of GUS activity was measured in transgenic plants of T_1_-T_3_ generations at the age of approximately 70 days. However, the dynamics of GUS activity in leaves of homozygous transgenic plants during the growth season is not known. In addition, the effect of the length of the light period on *pro-SmAMP2* promoter activity in heterologous plants was also not clear. As the *pro-SmAMP2* promoter contains regulatory elements potentially able to change its activity in dependence of the light condition (Fig. 1 and Table 1), we studied GUS activity under different light regimes.

**Fig. 6:**
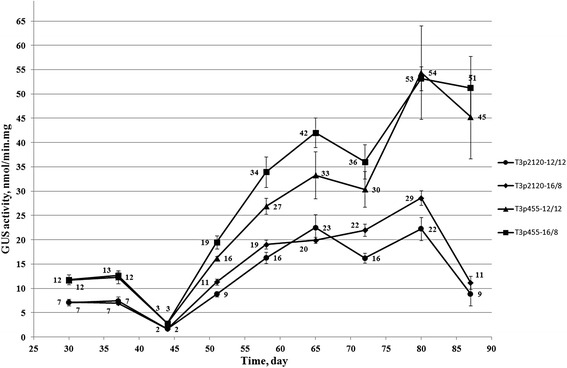
The GUS activity in the leaves of T_3_ generation homozygous transgenic tobacco plants grown in soil from seeds. Eight plants were studied under long day (16/8 h) and six plants under short day (12/12 h) conditions for both variants T_3_p2120 and T_3_p455. The average levels of GUS activity in adjacent leaves from medium tiers ± SE are presented

For this purpose, homozygous tobacco plants from transgenic lines Т_3_p2120 (No. 8-2-2 and No. 19-1-2) and Т_3_p455 (No. 14-3-3 and No. 10-8-9), were grown at short and long day, bypassing the selection stage on the medium with hygromycin. Data from Fig. 6 suggest that GUS activity was higher under long-day (16/8 h) than under short-day (12/12 h) condition on days 51, 58, 72 and 80 in Т_3_p2120 plants, and from day 51 to 65 in Т_3_p455 plants. However, these differences were not significant (*p* = 5 %), except for day 72 in Т_3_p2120.

Under long-day condition, GUS activity level was at least 1.5 times higher (*p* = 5 %) in Т_3_p455 than in Т_3_p2120 plants for the entire observation period (Fig. 6). The same trend was detected under short-day condition, except days 37 and 65 when the differences between Т_3_p455 and Т_3_p2120 were not significant (*p* = 5 %).

Figure 6 also shows that a GUS activity of 9–11 nmol/mg · min in Т_3_p2120 and 16–19 nmol/mg · min in Т_3_p455 plants, grown in the soil directly from seed, was reached on day 51, but not on day 70 as was observed for those plants, selected on the medium with hygromycin (Fig. 4с). In the subsequent days, GUS activity increased and reached on day 80 22–29 nmol/mg · min in Т_3_p2120 and 53–54 nmol/mg · min in Т_3_p455 (Fig. 6). In plants with the *pro-SmAMP2* promoter deletion variant −455 bp, GUS activity on day 80 was comparable to that previously measured in the primary transformants growing in soil (Fig. 3b). Measurements on day 87 showed that GUS activity decreased: more in Т_3_p2120 and less in Т_3_p455 plants (Fig. 6).

### GUS activity correlates with the content of *gus* gene mRNA in transgenic plants T_3_p2120 and T_3_p455

**Fig. 7:**
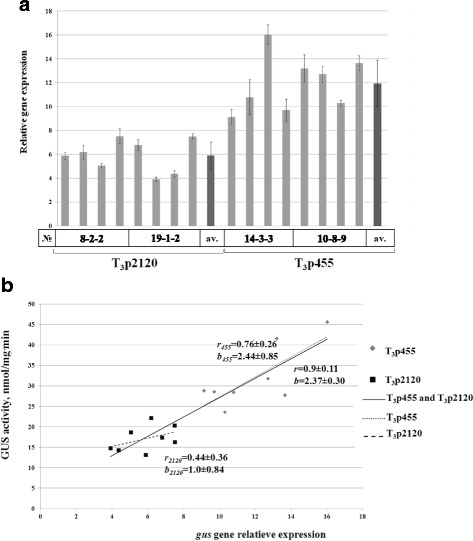
*Gus* gene expression in leaves of T_3_p2120 and T_3_p455 tobacco plants measured by real-time PCR. **a** *Gus* gene expression normalized to the expression of *actin* gene. Average values for individual transgenic plants ± SE are presented as gray bars. For transgenic variants, the average values ± confidence interval (*p* = 5 %) are presented as black bars. **b** The dependence of GUS activity level on *gus* gene expression. Correlation coefficients (*r*) and regression coefficients (*b*) ± SE are shown

The results of real-time PCR measurement of *gus* gene expression relative to the *actin* reference gene of tobacco are shown in Fig. 7a. It can be concluded from the data that: 1) *gus* expression was higher than *actin* gene expression in almost all transgenic plants, and 2) on average, *gus* gene mRNA content was two times higher in the samples from T_3_p455 than from T_3_p2120 plants.

Strong positive correlation (*r* = 0.9 ± 0.11) between GUS activity levels and *gus* gene mRNA expression was shown in the leaves of T_3_p455 and T_3_p2120 plant variants analyzed together as a pooled set (Fig. 7b). The correlation coefficient for T_3_p455 plants taken separately (*r*_*455*_ = 0.76 ± 0.26) also supported the strong relationship between *gus* gene mRNA content and the activity of its protein product, while the correlation was not that strong in the T_3_p2120 variant (*r*_*2120*_ = 0.44 ± 0.36).

When analyzed in a pooled group, activity of the GUS protein per unit of *gus* gene mRNA changed linearly with a regression coefficient of *b* = 2.37 ± 0.30 (Fig. 7b). This parameter did not differ significantly between separate T_3_p455 (*b*_*455*_ = 2.44 ± 0.85) and T_3_p2120 (*b*_*2120*_ = 1.0 ± 0.84) groups due to large statistical variation.

### *Pro-SmAMP2* promoter is active in different organs and at different stages of T_3_p2120 and T_3_p455 development

**Fig. 8:**
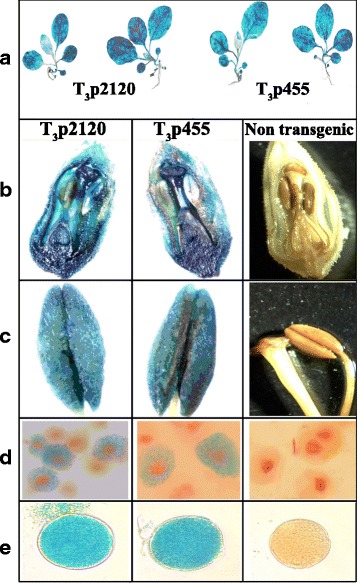
The GUS activity in different organs of Т_3_р2120 (line No. 8-2-2) and Т_3_p455 (line No. 10-8-9) transgenic tobacco plants at various stages of their development. **a** Roots, stems, and leaves at the age of 2 weeks after transplanting from aseptic conditions, (**b**) bud, (**c**) anthers, (**d**) microsporocytes, additionally stained by acetocarmine, (**e**) pollen

Staining showed that activity of both *pro-SmAMP2* promoter deletion variants is present in leaves, stems, roots, buds, anthers, microsporocytes and pollen. Particularly intense staining was noted in the hypocotyl of all transgenic plants, which indicates a higher activity of the *pro-SmAMP2* promoter region in this part of a plant (Fig. 8a). The least intense staining of leaves present in transgenes was typical for the youngest apical ones.

Histochemical analysis allowed us to conclude that the *pro-SmAMP2* promoter is active not only in plant cells at the stage of sporophyte but also during meiosis and at the stage of gametophyte, as GUS activity was detected in the microsporocytes and pollen (Fig. 8d, f).

At the same time, a high level of GUS activity in all studied organs and cells of transgenic plants did not allow to visually identify differences between the T_3_p2120 and T_3_p455 variants. Moreover, in some transgenic plant samples subjected to the histochemical analysis, products of X-Gluc substrate hydrolysis stained not only the plants themselves but also the incubation solutions, that indicates that the levels of GUS protein in these samples is very high. It was also noted that if the plant tissue was damaged, solution staining was more intense.

Quantitative method of GUS activity assay using 4MUG substrate was used for comparative analysis of *pro-SmAMP2* promoter deletion variants activity in T_3_ seeds obtained from T_2_p2120 and T_2_p455 tobacco plants. The activity from 1.2 to 19.8 nmol/mg^**.**^min was detected in T_3_ seeds, but it varied strongly even between plants from the same line. This finding was quite surprising, because in chickweed plants, the *pro-SmAMP2* gene promoter ensures the accumulation of antifungal peptides SmAMP1.1a and SmAMP2.2a in seeds [26].

## Discussion

In previous studies, we established that *pro-SmAMP2* gene expression in chickweed was at a very high level comparable with the level of *actin* gene expression, and the highest values were observed in the roots and flowers of plants [26]. In addition, *pro-SmAMP2* gene expression in aseptic chickweed plants increased from two to five times due to the contact with phytopathogenic fungi, as well as after methyl jasmonate treatment. The combination of these results suggests that the *pro-SmAMP2* gene promoter is strong, slightly inducible and organ-specific in chickweed. The properties of the *pro-SmAMP2* gene promoter region in chickweed plant are determined by two factors, namely, the presence of specific regulatory elements in its nucleotide sequence, as well as the presence and activity of respective transcription factors within the plant cells.

It is known that the promoters of PR-protein genes are usually strong [24], and the location of specific motifs such as CATTTCCACTATATATAG, CAAT-box, CATAAACATAAAC in the promoter core segment confirms this. Such motifs are found in promoters of plant genes with high expression level [31]. The nucleotide sequence of the *pro-SmAMP2* gene promoter region contains conserved elements of TATA-box, CAAT-box motif and the sequence of transcription initiation site (Fig. 1). In addition, the promoter also contains some less conserved elements which may serve to fine-tune gene expression in tissues, organs or in response to different stimuli (Table 1). Among the latter ones there are probably *cis*-regulatory elements that ensure the activation of *pro-SmAMP2* gene expression in aseptic chickweed plant after fungal infection or methyl jasmonate treatment.

Analysis of *gus* reporter gene expression under the control of various deletion variants of *pro-SmAMP2* gene promoter in aseptic transgenic tobacco plants allowed to identify the minimum length of −455 bp retaining promoter activity. The shorter deletion variant −290 bp did not show any activity in heterologous plants, despite the fact that they contained a TATA-box and CAAT-box. The loss of activity may be associated with the deletion of the GT1 motif located in the area from −455 to 290 bp (Fig. 1) which is necessary for the initiation and stabilization of the transcription complex TFIIA-TBP-TATA [32]. It is unlikely that the loss of another *cis*-element Skn1 located in the same area of promoter sequence causes inactivation of the promoter transcriptional activity, because this regulatory element is associated with storage protein gene expression in the endosperm of a seed but not in leaves [33].

The present study was performed using the strong and constitutive viral promoter *CaMV 35S* as a control. With this promoter, the level of GUS activity was below 1.8 nmol/mg.min in individual transformants of tobacco cultivar Samsun NN (Fig. 3). In general, this activity level is comparable with the results of other studies which reported the GUS activities of 0.9–3.3 nmol/mg · min in individual transgenic lines of Samsun NN or Xanthi tobacco cultivars [12, 30]. Meanwhile, there are studies which provide experimental data demonstrating GUS activity as high as 10 nmol/mg · min under *CaMV 35S* promoter in some tobacco lines [25]. It is known that the level of GUS activity is highly variable in transgenic tobacco plants when using the *CaMV 35S* promoter which probably depends on the promoter version or measurement conditions. Anyway, GUS activity level with *CaMV 35S* promoter demonstrated in those studies that the *CaMV 35S* promoter can be used as a reference to compare our results with the data of other published research.

In aseptic tobacco plants, deletion variants of *pro-SmAMP2* gene promoter region −1504, −1149 and −455 bp, but not −2120 bp, provided a significantly higher activity of the reporter protein than known viral *CaMV 35S* promoter (Fig. 3a). These results suggest that the *pro-SmAMP2* gene promoter will be strong in dicotyledonous plants in case of stable integration into the genome. It was surprising that the longest *pro-SmAMP2* promoter deletion variant −2120 bp was inferior to the other deletion variants and did not differ from *CaMV 35S* promoter in efficiency. This suggests that in the segment from −2120 to 1504 bp of *pro-SmAMP2* promoter region, there are regulatory elements acting negatively in aseptic tobacco plants. Likely candidates could be three TGACG motifs, which are responsible for gene expression effects of methyl jasmonate in barley *Hordeum vulgare* [34], and GT1 motif capable of affecting gene expression in both positive and negative way [35].

It was quite unexpected to observe an increase of GUS activity in transgenic T_0_ tobacco plants after their transplantation into soil (Fig. 3), and this effect was most prominent in the T_0_p2120 plants, but less pronounced and comparable in magnitude in T_0_p1504, T_0_p1149, and T_0_p822. In T_0_p455 plants, the increase in GUS activity was also noted, but compared to the other groups it was very small and only slightly higher than under viral promoter *CaMV 35S*. Based on these findings, we assume that the effect of expression induction could be mainly the result of the cumulative effect of TGACG elements [34], partly W box [36] and S box [37] *cis*-elements involved in the response to elicitors of pathogenic fungi. Association of soil microorganisms could act as inductor of *pro-SmAMP2* promoter region in transgenic tobacco plants in the conditions of greenhouse.

Previously, we have shown that the *pro-SmAMP2* gene has the highest expression level in roots and flowers in chickweed plants [26]. However, in transgenic tobacco T_0_p1504, T_0_p1149, T_0_p822, and T_0_p455, the highest GUS activity was observed in the leaves. This may be the result of formerly described cooperative interaction of *cis*-elements GT1 and I box (GATA-type light regulatory element), which are present in the core part of the *pro-SmAMP2* promoter, in response to light [38–41]. As tobacco plant leaves have larger surface area for light absorption than other organs, the maximum effect from GT1 and GATA motifs appeared, apparently, in them. Noteworthy, it has been demonstrated that the presence of GT element leads in some cases to the gene suppression in non-photosynthetic organs of plants [35], which correlates with our data, since GUS activity in roots of transgenic tobacco plants was significantly lower than in the above-ground parts.

The relatively high level of GUS activity was preserved in T_1_ transgenic tobacco plants with all deletion variants of the *pro-SmAMP2* promoter region (Fig. 4a). However, the absolute values of GUS activity were significantly reduced in all groups, except T_1_p455, and the values were closer to the level of GUS activity in T_0_ aseptic plants (compare Fig. 3b and 4a). The most dramatic decrease of activity was observed in variant T_1_p2120, whereas the reduction was less pronounced and comparable between T_1_p1504, T_1_p1149, and T_1_p822. The reason for the above activity changes is not clear; however, a certain similarity of this result with the above-described induction of *gus* expression in plants T_0_p2120, T_0_p1504, T_0_p1149, and T_0_p822 in the soil (Fig. 3b) suggests that the reduction in GUS activity may be associated with TGACG *cis*-elements involved in the response to methyl jasmonate [34].

Nevertheless, the *pro-SmAMP2* promoter efficiency in the progeny of transgenic T_1_ tobacco plants was comparable to the efficiency of the viral promoter *CaMV S35*, which can induce GUS activity in transgenic tobacco plants as high as 0.9–10.0 nmol/mg · min according to published data [12, 25, 30].

High GUS activity was preserved on the level of their parent plants in transgenic tobacco plants of generations T_2_ and T_3_ transformed by *pro-SmAMP2* promoter deletion variants −2120 and −455 bp (Fig. 4b and с). Individual T_3_ transgenic plants did not segregate on the selective medium with hygromycin and had a comparable level of GUS activity within individual populations, which suggests their homozygosity by T-DNA loci. The average level of GUS activity in all populations of Т_3_p455 was about 20 nmol/mg · min which was approximately two times higher than the activity in Т_3_p2120 (Fig. 4b and с). These results imply the presence of *cis*-elements in the region from −455 to 2120 bp of *pro-SmAMP2* promoter which reduce promoter activity in tobacco plants. At the same time, it is possible that these *cis*-elements may contribute to the induction of the *pro-SmAMP2* promoter in response to certain inducers; however, we failed to identify the inducers.

In homozygous transgenic tobacco plants T_3_p2120 and T_3_p455 grown from seed in the soil, the *pro-SmAMP2* promoter led to accumulation of the GUS reporter protein during the vegetative period from day 30 to 87 (Fig. 6). In T_3_p455 plants, but not in T_3_p2120, GUS activity on day 80 reached the level detected earlier in the primary transformants grown in the soil (Fig. 3b and 6). These results confirm the assumption that negatively acting *cis*-elements are present in the *pro-SmAMP2* promoter sequence region from 2120 to 455 bp.

More daylight hours led to increased GUS accumulation in both T_3_p455 and T_3_p2120 plants, which implies that *pro-SmAMP2* promoter activity positively depends on the duration of illumination. This was expected, since its nucleotide sequence contains *cis*-elements able to react to changes in the duration of the light period (Fig. 1 and Table 1). It should be noted that the *CaMV 35S* viral promoter in transgenic tobacco plants, on the contrary, had higher activity under short day condition (8/16 h) than under long day (16/8 h) [30].

Overall, we see that in successive generations, the T_1_-T_3_ GUS activity in transgenic tobacco plants with the shortest deletion variant −455 bp of *pro-SmAMP2* promoter was approximately 1.5 to 2-fold higher than in plants with the longest deletion variant of the promoter.

Quantitative measurement of *gus* mRNA showed that its level is much higher than the expression of the tobacco *actin* gene in many lines of T_3_ transgenic plants. The shorter deletion variant −455 bp of *pro-SmAMP2* promoter produced 2-fold higher expression level of *gus* mRNA than the longest deletion variant (Fig. 7a), what correlates with the GUS activity. This positive correlation between *gus* gene expression level and the activity of its protein product GUS (Fig. 7b) confirms that high GUS activity in transgenic tobacco plants T_3_p2120 and T_3_p455 is primarily related to the reporter gene transcription, but not to the translation of its mRNA, as was the case with *VR-ACS1* gene promoter from *Vigna radiata* L. [25]. It should be noted that all deletion variants of the *pro-SmAMP2* promoter include its own 40 nucleotide long 5′UTR (Fig. 1). It is quite possible that it positively affects the stability of *gus* mRNA, but in order to make this assertion more research is needed.

The regression coefficient *b* = 2.37 ± 0.30 was obtained for GUS activity per mRNA level unit calculated for all the transgenic tobacco plants on day 58 when grown under long-day condition (Fig. 7b), that indicates the process of intensive accumulation of GUS reporter protein. This assumption is supported by the dynamics of GUS activity in transgenic plants which is also intensively increasing from day 42 to 80 (Fig. 6). The active form of the GUS protein in plants is characterized by a long half-life time [42]. Therefore, the process of high accumulation of GUS protein in transgenic plants T_3_p2120 and T_3_p455 may be explained not only by transcriptional activity of *pro-SmAMP2* promoter but also by the integral effect of GUS accumulation.

In Fig. 8, it is demonstrated that the activity of the *pro-SmAMP2* promoter deletion variants −2120 and −455 bp, which are most different in length, is present in leaves, stems, roots, buds, anthers, microsporocytes and pollen of T_3_ transgenic plants. Particularly intense staining by the products of substrate X-Gluc hydrolysis was noted in the region of hypocotyl (Fig. 8a), suggesting increased activity of the *pro-SmAMP2* promoter in this part of the plant. These results can be helpful when creating transgenic plants resistant to pathogens that damage crops in the region of the hypocotyl (for example, tomatoes and flax).

GUS activity in seeds of the studied plants varied greatly, probably due to inactivation of GUS enzymatic activity during storage or due to seed dehydration. It should be noted that even in plants of the same line seeds were ripening with a 1 to 3 weeks difference.

## Conclusion

From the results obtained with transgenic tobacco plants, we have concluded that the *pro-SmAMP2* gene promoter is strong, and its most promising deletion variant for target gene expression in plants is a variant of −455 bp length. Given the high level of *gus* expression in transgenic tobacco plants under control of this promoter and the high level of *pro-SmAMP2* gene expression in *S. media* plants [26], it is expected that the *pro-SmAMP2* gene promoter will retain high activity in other dicotyledonous plants.

## Methods

### Cloning nucleotide sequence of *pro-SmAMP2* gene promoter region from chickweed *S. media*

The seeds of *S. media* plant were collected in Central Russia. Genomic DNA extracted from leaves by GenElute Plant Genomic DNA Miniprep Kit (Sigma, USA) was used as the source of *pro-SmAMP2* gene promoter region.

The nucleotide sequence of the *pro-SmAMP2* gene promoter was determined using the method of “genome walking”. This was done using Genome Walker Universal Kit (Clontech Laboratories Inc., USA) and two antisense primers 1 and 2 specific for *pro-SmAMP2* gene sequence (Additional file 1: Table S2).

The target nucleotide sequence was amplified by two-step PCR according to the instructions of the manufacturer. The PCR product 2400 bp long was cloned in the vector pGEM-T (Promega, USA) and sequenced.

### Producing genetic constructs for *Agrobacterium*-mediated plant transformation

The first nucleotide of the transcription start site (TSS) was designated as +1. Deletion variants of the *pro-SmAMP2* gene promoter region −2120, −1504, −1149, −822, −455 and −290 bp with 5′UTR (40 bp) were obtained (Fig. 1) using the reverse primer 3 and the corresponding direct primers 4–9 (Additional file 1: Table S2). For convenience of cloning, reverse primer 3 contained the sequence of the restriction site *Nco*I at its 5′-end, and direct primers 4–9 contained *EcoRI* site.

To prevent polymerase errors, PCR amplification was carried out with a mixture of *Pfu* (SibEnzyme, Russia) and *Taq* (Syntol, Russia) DNA polymerases in the ratio of 1:10 in the standard reaction buffer for thermostable polymerase (60 mM Tris–HCl (pH 8.5 at 25 °C); 1.5 mM MgCl_2_; 25 mM KCl; 10 mM 2-mercaptoethanol; 0.1 % Triton X-100) with dNTPs. The previously obtained 2400 bp fragment of the *pro-SmAMP2* gene promoter region was used as DNA template. Amplification profile: denaturation 94 °C, 30 s; primer annealing 60 °C, 40 s; elongation 72 °C, 2 min; 25 cycles.

Plasmid genetic constructs for plant transformation containing the reporter gene *gus* controlled by the deletion variants of *pro-SmAMP2* gene promoter region were obtained by merging DNA fragments with the coding region of the reporter gene *gusA* in the plasmid pCambia1381Z (Cambia, Australia). For this purpose, PCR amplicons were purified, treated with the appropriate restriction enzymes and ligated into plasmid pCambia1381Z at the restriction sites *Nco*I and *EcoR*I. All genetic constructs obtained were verified by sequencing and designated, respectively, p2120, p1504, p1149, p822, p445 and p290. As a control, plasmid pMOG-35SintGus was used in which *gus* reporter gene is placed under control of the viral promoter *CaMV 35S* [43].

### Agrobacterial strains for plant transformation

Plant expression vectors p2120, p1504, p1149, p822, p445, p290 and pMOG-35SintGus were introduced into cells of *Agrobacterium tumefaciens* strain AGL0 by electroporation. Agrobacterial transformants containing plant expression vectors were selected on agar LB medium with the addition of antibiotics kanamycin (PhytoTechnology Laboratories, USA) and rifampicin (PhytoTechnology Laboratories, USA) at a concentration of 100 mg/L each. For plant transformation, *Agrobacterium* strains were grown in LB liquid medium with rifampicin (100 mg/L) and kanamycin (100 mg/L) for 20 h at 27 °C and 180 rpm.

### Obtaining T_0_ generation transgenic plants

Tobacco plants *Nicotiana tabacum* cultivar Samsun NN were used for *Agrobacterium*-mediated transformation. Regenerants were produced according to previously published methodology [26]. The antibiotic timentin (PhytoTechnology Laboratories, USA) was used at a concentration of 300 mg/L for elimination of agrobacteria. For selection of regenerants transformed by plant expression vectors p2120, p1504, p1149, p822, p445 and p290, hygromycin (PhytoTechnology Laboratories, USA) in a concentration of 50 mg/L was used; for those transformed by the plasmid pMOG-35SintGUS, kanamycin was used (100 mg/L). The regenerants were designated according to the genetic structure used for transformation. Aseptic tobacco plants were grown at 23 °C, illumination 7 klx and photoperiod 16/8 h, then they were adapted to the soil within 3 days and were grown in the greenhouse at 26 °C, illumination 11 klx and 16/8 h photoperiod, unless otherwise stated.

### Obtaining T_1_–T_3_ generation transgenic plants

Seeds of T_1_-T_3_ were obtained by self-pollination of tobacco plants from preceding generations without obvious morphological abnormalities, demonstrating the expression of *gus* reporter gene and capability of seed formation. Seeds from each self-pollinated plant were collected separately, sterilized and selected on Murashige-Skoog medium [44] with hygromycin (50 mg/L) for 4 weeks. After selection was done, segregation analysis of plants into green and white phenotype was performed using Chi-square criterion. Green plants were adapted to the soil within 3 days, and were grown in the greenhouse at 26 °C, illumination 11 klx and the photoperiod 16/8 h.

To obtain homozygous plants of T_2_ generation, the T_1_ plants from populations with monogenic inheritance of transgenes were used to avoid the influence of the number of independent T-DNA insertions on GUS activity level. Plants with activity level approximately two times higher than in other plants of the same population were selected as the parent T_1_ plants, which suggested their homozygosity by T-DNA loci. The homozygous T_3_ plants were obtained on the same way.

### Quantitation of the reporter protein beta-glucuronidase (GUS) activity

GUS activity was measured in extracts of tobacco plants according to the method by Jefferson et al. [45]. Three tissue samples were analyzed from each individual transgenic plant. To obtain protein extracts, plant tissue samples (about 10–20 mg) were homogenized in 150 μL of extraction buffer (50 mM NaH_2_PO_4_ (pH 7.0); 10 mM EDTA; 0.1 % Triton X-100; 0.1 % (w/v) sodium laurylsarcosine; 10 mM beta-mercaptoethanol), centrifuged 10 min at 15.000 rpm, 4 °C. Next, 100 μL of supernatant was collected and re-centrifuged under the same conditions, and 70 μL of the supernatant was taken for analysis. The samples obtained were stored frozen at minus 70 °C.

GUS activity was measured for 30 min at 37 °C in 100 μL of extraction buffer with 4-methylumbelliferyl-D-glucuronide (4MUG) (PhytoTechnology Laboratories, USA) at a final concentration of 1 mM. The reaction was stopped by adding 900 μL of 0.2 M Na_2_CO_3_. Fluorescence was excited at 365 nm and measured at 455 nm (Perkin Elmer LS55, USA). Calibration of fluorescence was performed using a solution of 4-methylumbelliferone (4MU) (Sigma-Aldrich, USA) in 0.2 M Na_2_CO_3_. GUS activity was calculated after determining the concentration of protein in extracts by Bradford [46] using a solution of bovine serum albumin as standard. The results of GUS activity measurement from each individual transgenic plant are presented as average value from three tissue samples ± SE.

Each aseptic tobacco T_0_ regenerant was analyzed in 1.5 months after rooting the plant on the medium with a selective agent, immediately before planting in soil. Each T_0_-T_3_ transgenic tobacco plant was analyzed 6 weeks after transplanting from aseptic conditions into the soil. Mature leaves (about 20 cm) from medium level were used for the analysis. One 10–12 mg cutting was taken to measure GUS activity in the leaves. Fragments of sprout stems (8–10 cm) with a diameter of about 1 cm (15–20 mg) were used for GUS activity analysis in the stems of tobacco plants. GUS activity was analyzed in the flowers of tobacco plants during abundant flowering, entire flower was used for the analysis. Adventitious roots about 1 cm long (weight 15–20 mg) from 8 to 10 cm sprouts were used for GUS activity measurement in the roots of tobacco plants.

GUS activity level at different daylight duration was studied in the period from day 30 after T_3_ homozygous seeds germination until the first fruits ripening on day 87. The plants were grown directly from seed in the soil, bypassing selection stage on the selective medium with hygromycin, which had duration of 28 days in other experiments. Seven transgenic tobacco plants from each line were analyzed, three of them were grown under short-day (12/12 h) and the other four under long-day (16/8 h) condition.

### Histochemical staining of T_3_ plants using X-Gluc

Five plants from each population were studied: populations No. 8-2-2 and 19-1-2 for Т_3_р2120 transgene; populations No. 10-8-9 and 14-3-3 for Т_3_p455 transgene (Fig. 5a). Expression of the *gus* reporter gene in different organs (root, stem, leaf, bud, anthers) and at different stages of plant development (gametophytic and sporophytic) was studied using histochemical staining of tissues with GUS substrate X-Gluc.

Whole transgenic tobacco plants grown in soil for 2 weeks after transplanting from aseptic condition, or buds, anthers, microsporocytes and pollen during flowering and fruiting were used for staining. For detection of GUS activity staining was performed according to the method by Jefferson et al. [42]. Whole plants or their organs were fully immersed in the staining solution (10 mM EDTA; 0.1 % Triton X-100; 1 mM K_3_Fe(CN)_6_; 1 mM K_4_Fe(CN)_6_; 5-bromo-4-chloro-3-indolyl-β-D-glucuronic acid, sodium salt (X-Gluc) (PhytoTechnology Laboratories, USA) 2 mM in 0.1 M phosphate buffer, pH 7.0). Samples were exposed to vacuum (80 kPa) for 30 min, then incubated at 37°С for 24 h and washed in 70 % ethanol.

### RNA extraction and synthesis of first chain cDNA

Total RNA was extracted from plant leaves (100 mg) using Trizol reagent (Invitrogen, USA) according to the instructions of the manufacturer. To eliminate genomic DNA contamination, RNA was treated with DNase RQ1 RNase-Free (Promega, USA) and stored at minus 70 °C.

The first chain cDNA was obtained by reverse transcription using the oligonucleotide oligo-dT (Syntol, Russia) as a primer and RNA-dependent DNA polymerase of Malone mice leukemia virus (M-MLV). The final incubation mixture contained 50 mM Tris–HCl (pH 8.2), 8 mM MgSO_4_, 10 mM DTT, 50 mM KСl, 0.4 mM each of dNTPs, 100 pmol oligo-dT primer, 5 units of RNase inhibitor, 25 units of reverse transcriptase and 1 μg of total RNA. The reaction was carried out for 1 h at 37 °C.

### Real-time RT-PCR

Four transgenic plants of each population from each deletion variant Т_3_p2120 (No. 8-2-2 and No. 19-1-2) and Т_3_p455 (No. 14-3-3 and No. 10-8-9) at the age of 58 days from the moment of seed germination in the soil grown under long-day condition (16/8 h) were used to study correlation between GUS activity and *gus* gene mRNA content.

Evaluation of *gus* gene expression relative to the expression of *actin* gene was performed as triplicate using specific primers (Additional file 1: Table S3, except gus-1 and gus-2 primers). The primers were selected so that the length of the PCR product was less than 150 base pairs. Specificity of amplification was checked by electrophoresis in 1.5 % agarose gel; if single amplicon was observed, amplification was assumed to be specific. Additionally, to confirm the specificity of the primers used, amplification products were cloned in the vector pGEM-T and sequenced on an automated sequencing machine AhFexpress II (Amersham Pharmacia Biotech, USA). Real-time PCR was performed on a cycler CFX96 (BioRad, USA) in the presence of the intercalating dye SYBR Green I in 25 μL of reaction mixture containing 50 mM KCl, 10 mM Tris–HCl (pH 8.3), 1.5 mM MgCl_2_, 5 mM each of dNTPs, 1 unit of *Taq* DNA polymerase, 5 μM specific primers, and 0.01 μL of the reverse transcription product.

Real-time PCR was performed under the following cycling conditions: 1 cycle 94 °C, 3 min; 40 cycles of (94 °C, 15 s; 60 °C, 15 s; 72 °C, 30 s). Specificity of amplification was checked after the last PCR cycle by analysis of the melting curve (from 55 to 95 °C). Reaction efficiency was determined by performing real-time PCR analysis with several dilution steps of cDNA. Statistical processing of PCR data was performed using the program qgene-96 [47]. For expression analysis, ΔΔC_t_ method was applied [48].

To calculate the changes of gene expression in the test samples compared to control ones, the following equation was used: C = (1 + E)^- ΔΔCt^,where ΔΔC_t_ = ΔC_t_ (gus) - ΔC_t_ (actin), C_t_ (gus) - threshold cycle difference between test and control samples for *gus* gene, ΔC_t_ (actin) - threshold cycle difference between test and control samples for *actin* gene, E – reaction efficiency.

### Southern blot hybridization

Tobacco genomic DNA (20 μg) was digested overnight at 37 °C with 60 U *EcoR*I which cut the T-DNA of p2120 and p455 at a single position (see Producing genetic constructs for *Agrobacterium*-mediated plant transformation)*.* The fragments were separated on a 0.9 % agarose gel and transferred to a positive-charged nylon membrane Hybond N+ (GE Healthcare,UK) by capillary blotting following the manufacturer’s instructions. The DNA probe was constructed by PCR using plasmid p2120 as the template, and primers gus-1 and gus-2 (Additional file 1: Table S3). Probe DNA (740 bp) was labeled with alkaline phosphatase using Amersham Gene Image AlkPhos Direct Labelling and Detection System (GE Healthcare, UK). Prehybridization, hybridization (overnight at 60 °C) with alkaline phosphatase-labeled probe, and subsequent washings of the membrane were carried out according to the AlkPhos Direct Labeling System protocol. Detection was performed using CDP-Star detection reagent following the manufacturer’s directions (Amersham CDP-Star Detection reagent, GE Healthcare, UK).

### Ethics approval and consent to participate

Not applicable.

### Consent for publication

Not applicable.

### Availability of data and material

The dataset supporting the conclusions of this article is available in the GenBank repository (accession number KX196447).

## Additional files

Additional file 1: Table S1. The GUS activity in T_0_p290 and wild type tobacco plants. **Table S2.** Primers for cloning *pro-SmAMP2* gene promoter region and its deletion variants. **Table S3.** Primers used in the gene expression measurements and Southern blot hybridization. (DOC 67 kb) Additional file 2: Figure S1. Relative GUS activity in different organs of T_0_ transgenic tobacco plants. The activity of GUS in leaves was taken as 100 %. (TIF 418 kb)

## Abbreviations

4MU: 4-methylumbelliferone
4MUG: 4-methylumbelliferyl β-D-galactopyranoside
ARE: adenylate-uridylate-rich element
bp: base pair(s)
CaMV: cauliflower mosaic virus
dNTP: deoxynucleoside triphosphates
EDTA: ethylenediaminetetraacetic acid
GCN4: general control nonderepressible
GUS: β-glucuronidase
klx: kilolux
LB medium: Luria broth medium
LRE: light responsive element
mRNA: messenger ribonucleic acid
MYB: myeloblastosis
PCR: polymerase chain reaction
PR-protein: pathogenesis-related
SE: standard error
SmAMP: *Stellaria media* antimicrobial peptide
T-DNA: transfer deoxyribonucleic acid
UTR: untranslated region
X-Gluc: 5-bromo-4-chloro-3-indolyl-β-D-glucuronide
