# Supplementary material for: Novel strong promoter of antimicrobial peptides gene pro-SmAMP2 from chickweed (Stellaria media)
Source: BMC Biotechnol. 2016 May 18;16:43. doi: 10.1186/s12896-016-0273-x (PMC4870781; doi:10.1186/s12896-016-0273-x)
Supplement: Additional file 1: Table S1. — The GUS activity in T0p290 and wild type tobacco plants. Table S2. Primers for cloning pro-SmAMP2 gene promoter region and its deletion variants. Table S3. Primers used in the gene expression measurements and Southern blot hybridization. (DOC 67 kb) [file 12896_2016_273_MOESM1_ESM.doc]

Table S1. The GUS activity in T_0_p290 and wild type tobacco plants.

| **Group of plants** | **№ of plant** | **GUS activity, nmol/mg·min*** |
| --- | --- | --- |
| Wild type  (non transgenic) | 1 | 0.006 |
|  | 2 | 0.006±0.001 |
|  | 3 | 0.011±0.001 |
|  | 4 | 0.008±0.001 |
|  | 5 | 0.008±0.001 |
| T_0_p290 | 1 | 0.011±0.001 |
|  | 2 | 0.038±0.015 |
|  | 3 | 0.007±0.001 |
|  | 4 | 0.009 |
|  | 5 | 0.007±0.001 |
|  | 6 | 0.009 |
|  | 7 | 0.005±0.001 |
|  | 8 | 0.010 |
|  | 9 | 0.009±0.001 |
|  | 10 | 0.009±0.002 |
|  | 11 | 0.008±0.001 |
|  | 12 | 0.007 |
|  | 13 | 0.004±0.001 |
|  | 14 | 0.007±0.001 |
|  | 15 | 0.003 |
|  | 16 | 0.036±0.016 |
|  | 17 | 0.013±0.001 |
|  | 18 | 0.008±0.001 |
|  | 19 | 0.008 |
|  | 20 | 0.034±0.016 |
|  | 21 | 0.006±0.001 |
|  | 22 | 0.004±0.001 |

* - The average activity values of three samples from one leaf of each transformant ± SE are presented.

Table S2. Primers for cloning *pro-SmAMP2* gene promoter region and its deletion variants.

| **Variant** | **Primer #** | **Sequence (5′-3′)** |
| --- | --- | --- |
| 2400 | 1 | ctgtgtctgagcctggtaataagcct |
|  | 2 | gcctgcggcagtaggctcaatctcagaaa |
| Rev | 3 | agcccatggtttcacttgatttttagtgtgactagt |
| -2120 | 4 | acggaattcgtcgcagatatataaacactccc |
| -1504 | 5 | acggaattcgcgactttttcacgttgcct |
| -1149 | 6 | acggaattcgacaaatttcactaacgaaaaaaaaaa |
| -822 | 7 | acggaattcgtaccaacgtgagtaactat |
| -455 | 8 | acggaattcggtatgttgtcacaataact |
| -290 | 9 | acggaattctctatagcctttatcttatc |

Table S3. Primers used in the gene expression measurements and Southern blot hybridization.

| **Gene** | **Primer** | **Sequence (5′-3′)** | **Amplicon, bp** |
| --- | --- | --- | --- |
| *gus* | fw | GTGTATATGAGGCTGATGATGG | 90 |
|  | rv | GAACTGGAACTGGAGATGACC |  |
| *actin* | fw | CTGGAATTGCTGATAGGATGAG | 111 |
|  | rv | AACCTCCAATCCAAACACTATAC |  |
| *gus* | gus-1 | tcgtaattatgcgggcaacgtc | 740 |
|  | gus-2 | cgaatcctttgccacgcaag |  |
