# Supplementary figures and images for: Novel strong promoter of antimicrobial peptides gene pro-SmAMP2 from chickweed (Stellaria media)
Source: BMC Biotechnol. 2016 May 18;16:43. doi: 10.1186/s12896-016-0273-x (PMC4870781; doi:10.1186/s12896-016-0273-x)

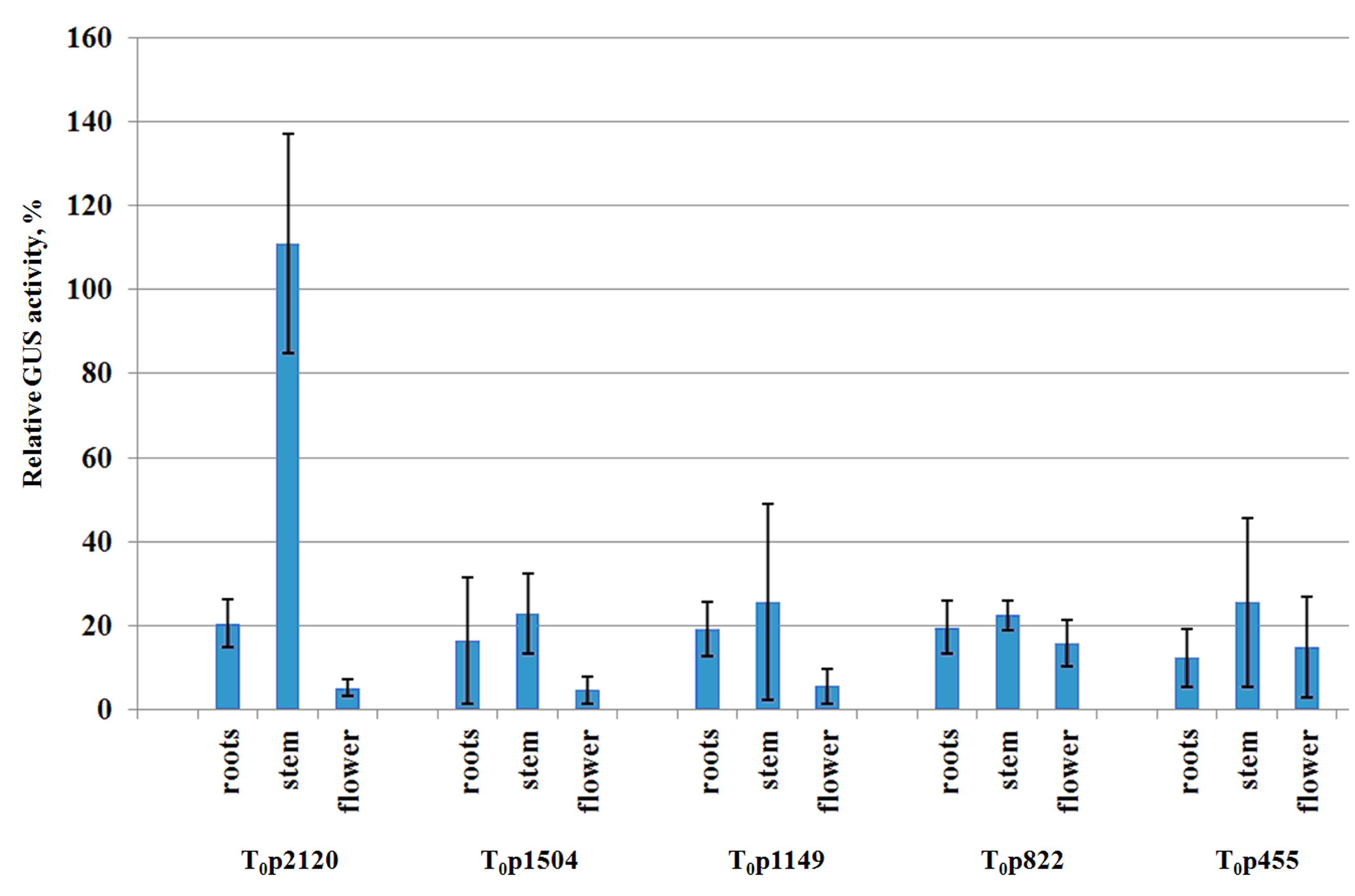

Supplement: Additional file 2: Figure S1. — Relative GUS activity in different organs of T0 transgenic tobacco plants. The activity of GUS in leaves was taken as 100 %. (TIF 418 kb) [file 12896_2016_273_MOESM2_ESM.tif]
